# Spontaneous Recanalization after Vasectomy

**DOI:** 10.1100/tsw.2006.367

**Published:** 2006-03-07

**Authors:** Antônio Marmo Lucon, Fábio Firmbach Pasqualotto, Edison Daniel Schneider-Monteiro, Luis Balthazar Saldanha, Alexandre Danilovic

**Affiliations:** ^1^ Division of Urology, University of São Paulo, Brazil; ^2^ Division of Urology and Pathology, University of São Paulo, Brazil

**Keywords:** vasectomy, infertility, failure, sterile, sperm

## Abstract

Vasectomy is the method most commonly used in men for voluntary sterilization purposes. We report two cases of early recanalization following vasectomies performed in 1085 men for sterilization purposes at a tertiary public institution between January 2000 and November 2003. Thus, the risk of 0.2% of failure due to early recanalization should be explained and the fertility implications stressed. Written documentation recording the clarification presented at consultation is essential.

## INTRODUCTION

Of all the methods of contraception, vasectomy is the safest, easiest, cheapest, most effective, and most reliable. It is usually uncomplicated; the discomfort after surgery usually abates promptly and there are generally no sequelae. Further, few patients suffer from bleeding or infection at the vasectomy site. Testicular pain is the most common complication after vasectomy. Although these complications can be serious, the usual conservative management leads to spontaneous cure[1].

Vasectomy may fail to result in sterility for three reasons, i.e., by failure to divide either vas, by vasal recanalization caused by the formation of microchannels between the severed ends, and by unrecognized anatomic variation.

Although definitions vary among investigators, early failure of the procedure is considered to have occurred when significant numbers of spermatozoa or any motile spermatozoa persist continuously later than 4 months after vasectomy[2]. Late failure may be defined as the presence of motile sperm some time after two consecutive azoospermic samples[1].

The purpose of our study is to report our experience with vasectomy spontaneous recanalization among 1085 patients treated with vasectomy for sterilization purposes.

## METHODS

Between January 2000 and November 2003, 1085 patients were submitted for vasectomy for sterilization purposes at a tertiary public institution. All patients were over 25 years old or had two or more children. Our institution has a standard protocol for the performance of vasectomy after referral that includes counseling and informed consent. Vasectomy procedure was performed under local anesthesia by a single physician (EDSM). Two 1-cm transversal hemiscrotal incisions were made. A 1-cm vas deferens segment was excised, followed by ligature, but not cauterization, of the cut ends using polypropilene 4-0 transfixed suture. Interposition of fascia was not done. Although the segments excised were not sent for pathological confirmation, the surgeon confirmed it to be vas deferens by passing a suture through its lumen. After vasectomy, patients were advised to maintain their use of contraceptives until they were considered sterile. Sperm samples were obtained before and 2 months after the procedure, and at the 4th month if the result was other than azoospermia.

## RESULTS

Vasectomy early recanalization occurred in 2 out of 1085 patients (0.2%). The first patient was 40 years old, his wife was 38, and they had four children. The second was 34 years old, his wife 28, and they had two children. A seminal sample was obtained from each patient before the vasectomy procedure and two additional samples were taken 2 and 4 months, respectively, after the surgery. The data of the sperm analysis are summarized in Table 1. However, as spermatozoids were observed in the postoperative control samples, these patients were submitted to surgical revision. Resection of both vasa was confirmed in both patients during the second procedure by exploration of the whole length of the vas deferens and visualization of the sutured cut ends involved in scar tissue, which was sent for histological study. The specimen on the first patient's right side and on the second patient's left side showed recanalization (Fig. 1).

**TABLE 1**
**Sperm Concentration and Motility Found in Semen Analysis of Patients with Spontaneous Vas Recanalization**

| | Before Vasectomy | | 2 Months after Vasectomy | | 4 Months after Vasectomy | |
|---|---|---|---|---|---|---|
| | Sperm Concentration ($\times 10^6$/ml) | Motility (%) | Sperm Concentration ($\times 10^6$/ml) | Motility (%) | Sperm Concentration ($\times 10^6$/ml) | Motility (%) |
| Case 1 | 225 | 85 | 10.6 | 56 | 18.5 | 65 |
| Case 2 | 128 | 75 | 70.1 | 48 | 37.9 | 78 |

## DISCUSSION

Early recanalization was first described in 1969 and is recognized by the fact that the first postvasectomy sperm count may be zero or greatly reduced and then increase rapidly[3]. Our two patients presented here experienced an important drop (more than 50%) of the first postvasectomy sperm count. While patient 1 had an increase in sperm count in the 4th month postvasectomy, patient 2 presented a further decrease in sperm count (Table 1). The outcome of these patients could be explained by a failure to remove at least one vas or an anatomic variation, nevertheless exploration of the whole length of the vasa and histological examination of the excised tissue in the second surgery showed the formation of one or more epithelialized channels through scar tissue in both patients (Fig. 1). These findings confirmed that the vasectomy failure was caused by early recanalization. After the second surgery, both patients were azoospermic in the 2nd and 4th month postsurgery. The etiology of this rare event is unknown, but it may be associated with sperm granulomas. Hanley suggested that the epithelial surface elements were responsible for failures because of the ability of these cells to create microtubules[3].

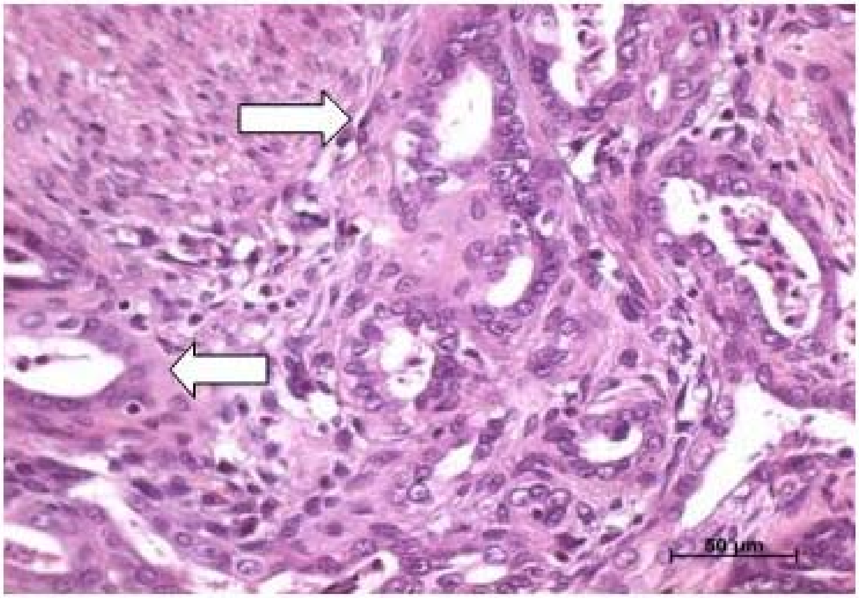

**FIGURE 1.** Recanalization of the vas deferens demonstrated by small ducts surrounded only by epithelial cells without a muscle layer.

Sperm that stagnate in the seminal vesicles are unlikely to retain normal structure, so it can be assumed that the presence of sperm with a normal structure, whether motile or not, indicates that recanalization has occurred[2]. Nonmotile sperm have been reported as having no functional significance and no confirmed associated pregnancies have been reported so far[2].

Late recanalization became recognized after six such failures were reported in 1984 and presented with unexpected pregnancies some time after clearance after two consecutive azoospermic samples. The chance of this occurring has been estimated to be 1 in 2900 (0.03%)[1]. Although the risk is slight, it is not therefore negligible, and failure to have warned the patient of this possibility in any vasectomy performed after 1984 has proved difficult to defend in a court of law. It is wise to warn the patient that the decision to discontinue contraception should be his rather than the doctor's[1]. Nowadays, there is no method to predict spontaneous recanalization after vasectomy; therefore, it is important to make clear that there is a small risk of pregnancy even when azoospermia has been demonstrated.

Because of the possibility of recanalization, different techniques have been described with a view to avoiding this complication[1]. Interposition of the fascial sheath between the severed ends of the vas reduced the recanalization rate to zero. Fulguration of the lumen of the cut vas ends was used, but was not the significant factor in this zero recanalization rate. The section fulguration-fascial interposition technique of vasectomy has been uniformly effective, with few postoperative problems. Failure, defined as either a pregnancy or the persistence of sperm in the semen, was not detected during follow-up[4].

A substantial increase in the failure rate was observed when the lengths of the excised vasa were less than 14–16 mm, which represents the critical minimum distance necessary to minimize the failure percentage. Segments with hemorrhage in tissue attached to the vas or large amounts of connective tissue surrounding the vas are associated with a lower percentage of successful operations[5].

## CONCLUSION

The risk of recanalization and its implications should be explained to the patient.

## ACKNOWLEDGMENTS

To Professor Sami Arap, for making possible the good outcome of this work.
